# Mannose receptor-mediated delivery of moss-made α-galactosidase A efficiently corrects enzyme deficiency in Fabry mice

**DOI:** 10.1007/s10545-015-9886-9

**Published:** 2015-08-27

**Authors:** Jin-Song Shen, Andreas Busch, Taniqua S. Day, Xing-Li Meng, Chun I. Yu, Paulina Dabrowska-Schlepp, Benjamin Fode, Holger Niederkrüger, Sabrina Forni, Shuyuan Chen, Raphael Schiffmann, Thomas Frischmuth, Andreas Schaaf

**Affiliations:** Institute of Metabolic Disease, Baylor Research Institute, 3812 Elm Street, Dallas, TX 75226 USA; Greenovation Biotech GmbH, Freiburg, Germany; Baylor Institute for Immunology Research, Dallas, TX 75204 USA; Baylor Research Institute, Dallas, TX 75226 USA

## Abstract

Enzyme replacement therapy (ERT) is an effective treatment for several lysosomal storage disorders (LSDs). Intravenously infused enzymes are taken up by tissues through either the mannose 6-phosphate receptor (M6PR) or the mannose receptor (MR). It is generally believed that M6PR-mediated endocytosis is a key mechanism for ERT in treating LSDs that affect the non-macrophage cells of visceral organs. However, the therapeutic efficacy of MR-mediated delivery of mannose-terminated enzymes in these diseases has not been fully evaluated. We tested the effectiveness of a non-phosphorylated α-galactosidase A produced from moss (referred to as moss-aGal) in vitro and in a mouse model of Fabry disease. Endocytosis of moss-aGal was MR-dependent. Compared to agalsidase alfa, a phosphorylated form of α-galactosidase A, moss-aGal was more preferentially targeted to the kidney. Cellular localization of moss-aGal and agalsidase alfa in the heart and kidney was essentially identical. A single injection of moss-aGal led to clearance of accumulated substrate in the heart and kidney to an extent comparable to that achieved by agalsidase alfa. This study suggested that mannose-terminated enzymes may be sufficiently effective for some LSDs in which non-macrophage cells are affected, and that M6P residues may not always be a prerequisite for ERT as previously considered.

**Electronic supplementary material:**

The online version of this article (doi:10.1007/s10545-015-9886-9) contains supplementary material, which is available to authorized users.

## Introduction

Lysosomal storage diseases (LSDs) are a group of life-threatening inherited disorders; most are caused by deficiency of a single lysosomal enzyme or protein, which leads to accumulation of substrate in cells. Currently, enzyme replacement therapy (ERT) is the principal specific treatment for several LSDs. Traditionally, the recombinant enzymes used in ERT are produced in cultured mammalian cells. Recently, as an alternative approach, plant-based expression systems have been utilized to produce lysosomal enzymes for therapeutic use (Shaaltiel et al 2007; Du et al 2008; He et al 2012). Relative to mammalian cell-based systems, plant-based systems have several advantages including lower production costs, eliminated risk of contamination by mammalian pathogens and, in the case of moss, a relatively easier manipulation of the N-glycosylation pathway. However, a major concern when considering using plant cell-produced enzymes for ERT is their N-glycan structures that usually differ from mammalian cell-produced enzymes. Particularly, lysosomal enzymes expressed in plant cells typically do not acquire mannose 6-phosphate (M6P) modification on terminal mannose residues (Gomord and Faye 2004).

Intravenously administered lysosomal enzymes are taken up by tissues through cell surface receptors that recognize the carbohydrate structure of the enzymes. M6P receptor (M6PR) and mannose receptor (MR) represent two major contributors to this uptake system. M6PR recognizes phosphorylated terminal mannose residues (M6P) and is expressed in most cell types (Kornfeld 1992). It is generally believed that in ERT used for most LSDs the M6PR-mediated endocytic pathway is crucial for sufficient enzyme delivery (Sands et al 2001; Sly et al 2006). On the other hand, MR recognizes terminal mannose, fucose and N-acetylglucosamine (GlcNAc) residues of glycoproteins (Stahl and Ezekowitz 1998). It was initially thought that the expression of MR is restricted to tissue macrophages, but now it is known that MR is also expressed in many other cell types including dendritic, endothelial, smooth muscle, and kidney mesangial cells (Stahl and Ezekowitz 1998). Mannose-terminated enzymes are thought to be effective in LSDs that affect macrophages, such as Gaucher disease (Barton et al 1991). Previous studies also demonstrated macrophage-targeted delivery of mannose-terminated protective protein/cathepsin A (PPCA), neuraminidase, and lysosomal acid lipase in animal models (Bonten et al 2004; Du et al 2008). However, the therapeutic efficacy of MR-mediated enzyme delivery in LSDs in which parenchymal (non-macrophage) cells are affected has not been fully evaluated.

In this study we addressed this question in Fabry disease, a glycosphingolipidosis caused by deficient activity of α-galactosidase A (α-gal A)(Brady et al 1967). As a result of the enzymatic defect, glycosphingolipids with terminal α-D-galactosyl residues, predominantly globotriaosylceramide (Gb_3_), accumulate in virtually all organs. Fabry disease exhibits a variety of clinical manifestations, of which stroke, cardiac dysfunction, and renal impairment are the most life threatening (Desnick et al 2001). Currently, two recombinant α-gal A preparations, agalsidase beta and agalsidase alfa, are used for ERT for Fabry disease (Eng et al 2001a, b; Schiffmann et al 2001). Both are produced from mammalian cells and contain M6P (Lee et al 2003). ERT with these enzymes is effective in reducing glycolipids accumulation in tissues, and appears to slow progression of the disease (Pastores 2007). In general, however, ERT does not produce completely satisfactory results when initiated in adults. Further investigations, including development of new forms of the enzyme and a better understanding of the mechanism of enzymatic uptake, are needed to improve the therapeutic outcome of ERT for this disease. In this study, we tested the enzymology, pharmacokinetics and pharmacodynamics of a new form of α-gal A in patient-derived cell lines and a mouse model of Fabry disease. This enzyme was produced from moss and contains mannose-terminated N-glycans with no M6P.

## Materials and methods

### Expression strain construction

The DNA encoding human α-gal A (NCBI Reference: NM_000169.2) was synthesized as a codon-optimized (for *Physcomitrella patens*) version and sub-cloned into a moss expression vector.

To generate α-gal A-producing moss cell lines, protoplasts of a moss double-knockout line devoid of plant specific α-1,3-fucose and β-1,2-xylose residues on its N-glycans (Koprivova et al 2004) were transformed with the expression cassettes by a PEG-based method and were selected using G418. More than 2000 drug-resistant moss plantlets were screened for total moss-aGal accumulation per biomass, and the strain with the highest expression was chosen as the production strain (for moss-aGal).

To produce α-gal A with increased numbers of terminal mannose (high-mann moss-aGal), the above production strain was further transformed with a knockout construct targeting N-acetylglucosaminyltransferase I gene.

### Enzyme production and purification

The moss-aGal production strain was cultivated for 4 weeks in a 20 L disposable bag placed in a Wave™ Reactor Rocker. At the end of cultivation, the culture broth was clarified and concentrated. Moss α-gal A enzymes were purified by three chromatographic steps (Butyl-650 M, DEAE, S) and were concentrated to ~0.5 mg/ml. For details see Supplementary materials.

### Glycan analysis

See Supplementary materials.

### Enzyme assay

α-Gal A activity was measured by the fluorimetric method as described (Durant et al 2011).

### Michaelis–Menten kinetics

See Supplementary materials.

### In vitro thermostability

See Supplementary materials.

### In vitro uptake study

Fabry patient-derived skin fibroblasts (DMN96.125) and endothelial cell line (IMFE1)(Shen et al 2007) were cultured in 10 % FBS in DMEM and EGM-2MV (Lonza) respectively. The cells were incubated with α-gal A preparations (10 μg/ml) in the presence or absence of inhibitors for indicated time lengths. After that, cells were harvested (by trypsin treatment that also eliminates extracellular α-gal A) for enzyme assay or immunoblot. For binding studies, IMFE1 cells were incubated with enzymes for 3 h at 4 °C. Then, the cells were washed with ice-cold PBS and directly lysed in 0.2 % Triton for α-gal A assay.

In uptake/binding studies, we used enzyme concentration of 10 μg/ml, which is in the range of theoretical maximum plasma concentrations of agalsidase alfa and beta (~5 and 20 μg/ml respectively) in infused patients receiving approved doses. A pilot study showed that uptake of both moss-aGal and agalsidase alfa in IMFE1 cells was dose-dependent up to 40 μg/ml (Supplementary Fig. 1), indicating that 10 μg/ml is below the saturation concentration.

### SDS-PAGE and western blot

See Supplementary materials.

### Immunofluorescence of cultured cells

See Supplementary materials.

### Animals and procedures

Both knockout Fabry mice (Ohshima et al 1997) and WT controls used in this study have mixed genetic background of C57BL/6J and 129 strains with ~75 % of C57BL/6J strain background (Shen et al 2015). Fabry mice accumulate Gb_3_ in most organs, thus mimicking Fabry disease biochemically. Preclinical ERT studies in Fabry mice permitted evaluation of the pharmacokinetics, biodistribution and dose-dependent substrate clearance of infused α-gal A (Ioannou et al 2001). Besides proof-of-concept, these data provided important information for determining the dosing regimen in clinical trials. Although there is only limited information for biodistribution of the enzyme in patients, it appears that plasma clearance, cellular localization, and tissue half-life of infused α-gal A in humans (Schiffmann et al 2000; Eng et al 2001a, b) are similar to those in Fabry mice (Ioannou et al 2001; Murray et al 2007). In addition to a number of ERT studies, Fabry mice have also been proven to be an invaluable preclinical model to test biochemical and functional correction of Fabry disease by gene therapy and substrate reduction therapy (Ziegler et al 2007; Ashe et al 2015).

All animal procedures were reviewed and approved by the Institutional Animal Care and Use Committee of Baylor Research Institute. Female Fabry mice (3–6 months old) were used throughout the study. For Gb_3_ clearance studies, female Fabry mice are better suited than male Fabry mice (Durant et al 2011), because male mice have testosterone-induced Gb_3_ synthesis in kidneys that confounds the effect of the infused enzyme in degrading accumulated Gb_3_. For all the injections, enzymes were diluted in saline to a total volume of 200 μl per mouse and were injected via tail-vein. Because of the limited availability of agalsidase beta, only agalsidase alfa was used in animal studies for comparison with moss-aGal.

### Pharmacokinetics

Enzyme preparations were injected at a dose of 1 mg/kg body weight (BW) (*n* = 5). Blood samples were collected by tail bleed at indicated time points. Plasma was separated for enzyme assay.

### Biodistribution and tissue kinetics

Enzymes were injected at 1 mg/kg BW. At indicated time points, mice were perfused with saline (to remove blood), and heart, kidneys, spleen, and liver were harvested. The whole organs were homogenized in 0.2 % Triton/saline for enzyme assay.

### Immunohistochemistry

Heart and kidney were harvested 1 day after enzyme infusion (1 mg/kg). Immunohistochemistry for human α-gal A was performed using a rabbit polyclonal antibody (see Supplementary materials).

### Clearance of tissue Gb_3_

Six-month-old female Fabry mice were injected with enzymes at doses of 0.3, 1, and 3 mg/kg BW. Heart, kidney, and liver were harvested 1 week after a single injection. Tissue Gb_3_ levels were analyzed by mass-spectrometry as described (Durant et al 2011).

### Statistical analysis

Data were presented as mean ± SEM. Statistical significance was determined by the Student’s *t*-test.

## Results

### Production and purification of moss enzymes

Human α-gal A was stably overexpressed in the moss *Physcomitrella patens*. The α-gal A expressing moss strain was a glycoengineered variant devoid of α-1,3-fucose and β-1,2-xylose residues on its N-glycans that are plant-specific and may elicit immunogenic response in mammals (Koprivova et al 2004). In the expression construct, N-terminal signal sequence of human *GLA* gene was replaced by a plant secretory signal peptide which is removed by signal-peptidase upon translation. The construct does not contain C-terminal vacuolar targeting sequence, which is often added to the protein of interest in plant expression systems to achieve mannose-terminated N-glycans. Therefore, the protein sequence of secreted moss-aGal is identical to the native human α-gal A (with signal peptide removed).

Production of moss-aGal was accomplished in a photoautotrophic fermentation process. The moss was grown in a pure mineral culture medium without any antibiotics or animal-derived component. Because of the lack of C-terminal vacuolar signal, moss-aGal is secreted into the culture medium instead of being sorted to the vacuoles. Recombinant α-gal A secreted in the medium was purified by column chromatography with a typical recovery rate of 30 %. The purified moss-aGal had 99 % purity by size exclusion chromatography and contained low levels (~100 ppm) of host cell proteins.

To test the effect of the increased number of terminal mannosyl residues on cellular uptake of the enzyme, α-gal A was also produced in a strain that was genetically depleted of its β-1,2-N-acetylglucosaminyltransferase activity. This knockout-modification results in an incapability of the moss to perform any complex-type glycan processing, as all later enzymatic steps lack their substrate. Hence, α-mannosidase I-mediated trimming in the cis-Golgi is the last processing step and therefore all N-glycans of this strain are of the high-mannose type. Human α-gal A produced in this strain is referred to as high-mann moss-aGal.

### In vitro characterization

Moss-aGal had very uniform N-glycans with core-type Man_3_GlcNAc_2_ as dominant structure (Table 1). Carbohydrate chains of moss-aGal were almost exclusively constituted of mannose and GlcNAc; both are MR ligands. In comparison, Man_5_GlcNAc_2_ was the most abundant glycan structure in high-mann moss-aGal (Table 1) with some small amounts of Man_4_, Man_6_, and Man_7_. There were no phosphorylated glycans in either moss-aGal or high-mann moss-aGal. Agalsidase alfa showed highly heterogeneous glycan structures, of which ~24 % were phosphorylated, ~7 % were mannose-terminated, and 63 % were diverse structures.

**Table 1:**
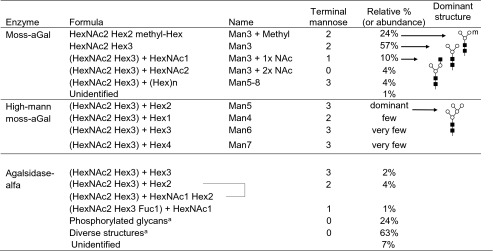
N-glycan analysis of the enzymes

N-glycans of moss-aGal and agalsidase alfa were analyzed quantitatively by HILIC-UPLC-MS

N-glycans of high-mann moss-aGal were analyzed as corresponding glycopeptides by ESI-Q-TOF mass spectrometry

Symbols in glycan structures: (*white circle*) mannose; (*black square*) GlcNAc; m, methyl group

^a^Detailed data are available upon request

**Fig. 1:**
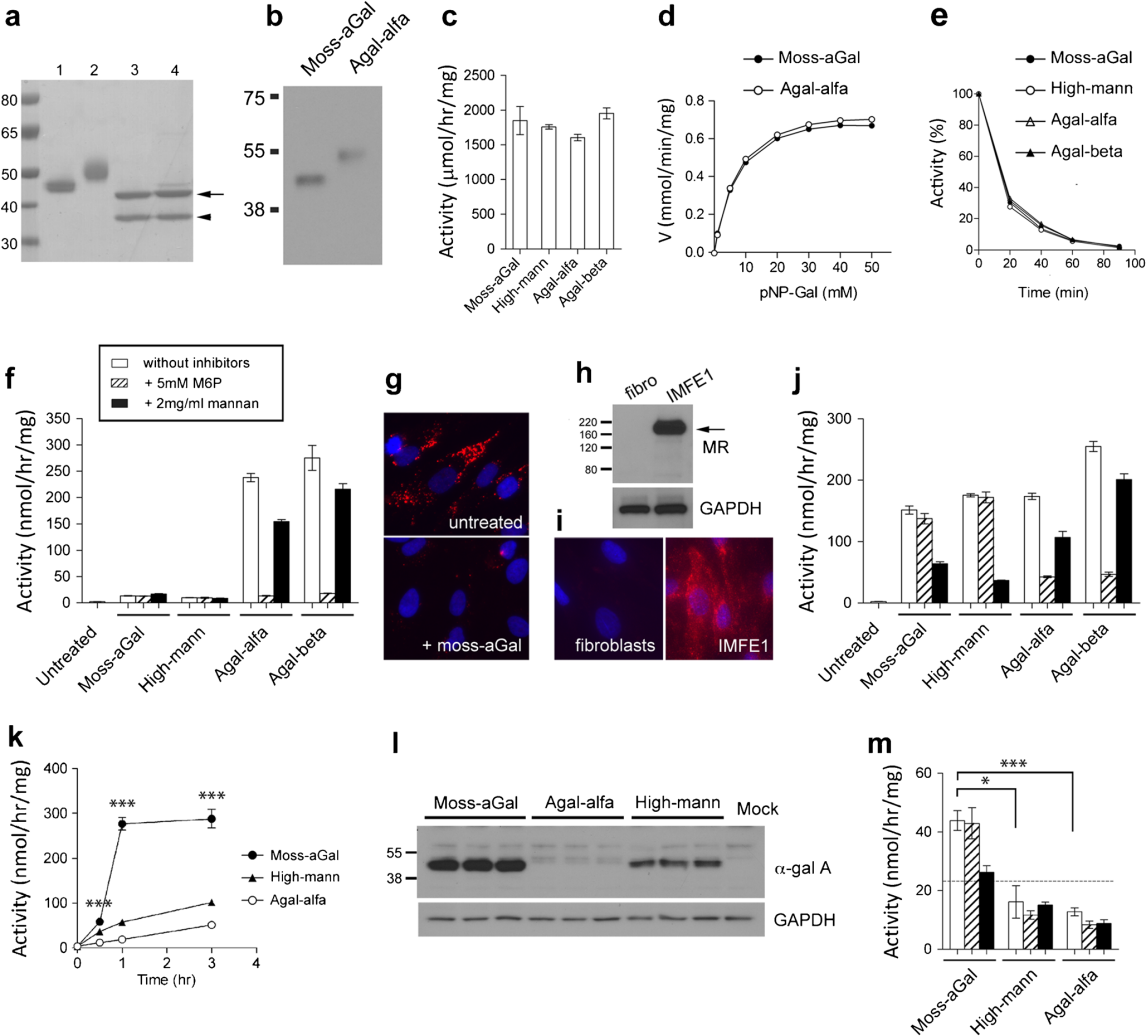
In vitro characterization and uptake studies **a** Enzyme preparations separated in SDS-PAGE and stained with Coomassie blue. Lanes 1 and 2 are moss-aGal and agalsidase alfa respectively. Lanes 3 and 4 are moss-aGal and agalsidase alfa digested with PNGase F. *Arrow*, α-gal A enzymes after digestion; *arrowhead*, PNGase F (36 KDa). Protein standard and molecular weights are shown on left. **b** Moss-aGal and agalsidase alfa (1 ng each) detected by western blot using a polyclonal antibody specific to human α-gal A. Representative data from three independent experiments was shown. **c** Specific α-gal A activities of enzyme preparations determined using artificial substrate 4-MU-α-D-galactopyranoside. **d** Plots of reaction velocities of moss-aGal and agalsidase alfa assessed with artificial substrate 4-nitrophenyl α-D-galactopyranoside (pNP-Gal). **e** Stability of the enzymes diluted in buffered human plasma and heated at 37 °C (data are means of triplicates). **f** Intracellular α-gal A activities of Fabry patient fibroblasts after overnight incubation with different enzymes in the presence or absence of 5 mM M6P or 2 mg/ml yeast mannan. **g** Gb_3_ immunofluorescence staining shows massive lysosomal accumulation of Gb_3_ in untreated Fabry patient fibroblasts (*upper*) and significantly decreased Gb_3_ in the cells that were treated with moss-aGal (*lower*). **h** and **i** MR expression in Fabry patient fibroblasts and microvascular endothelial cells IMFE1. IMFE1 cells were MR-positive determined by both western blot (**h**) and immunofluorescence staining (**i**), while the fibroblasts were MR-negative. **j** Intracellular α-gal A activities of IMFE1 cells after overnight incubation with different enzymes in the presence or absence of M6P or mannan. **k** Uptake rates of different enzymes in IMFE1 cells. Cells were harvested at indicated time points and intracellular activities were measured. ***P < 0.001, moss-aGal vs. high-mann moss-aGal or agalsidase alfa. **l** Western blot analysis of internalized α-gal A in IMFE1 cells after 3 h incubation with different enzyme preparations. **m** Binding of different enzymes (10 μg/ml) to IMFE1 cells. After 3 h incubation at 4 °C, cell surface-bound enzymes were determined by enzyme assay. The *dotted line* indicates activity level of mock-treated IMFE1 cells in this assay (i.e., background level). *P < 0.05, ***P < 0.001. All the data in graphs are presented as mean ± SEM (*n* = 3-4). High-mann: high-mann moss-aGal; Agal-alfa: agalsidase alfa; Agal-beta: agalsidase beta

In SDS-PAGE, moss-aGal was detected as a single major band with a faster mobility than agalsidase alfa (Fig. 1a), reflecting the lower carbohydrate content in moss-aGal. After removal of N-glycans by PNGase F, both moss-aGal and agalsidase alfa migrated to the same position (Fig. 1a). In western blot, both moss-aGal and agalsidase alfa were detected by a polyclonal antibody to human α-gal A (Fig. 1b). With the same amount of protein loaded, the intensity of moss-aGal band in immunoblot was 2.14 ± 0.58 times (*n* = 3) that of agalsidase alfa.

Specific activities of moss-aGal and high-mann moss-aGal were similar to those of agalsidase alfa and beta (Fig. 1c). Enzyme kinetics of moss-aGal assessed with artificial substrate 4-nitrophenyl α-D-galactopyranoside was almost identical to that of agalsidase alfa (Fig. 1d). Km of moss-aGal and agalsidase alfa were 6.5 ± 0.2 and 6.8 ± 0.3 mM; and Vmax of these enzymes were 0.78 ± 0.007 and 0.82 ± 0.008 mmol/min/mg, respectively.

Moss-aGal and high-mannose moss-aGal had almost the same stability as agalsidase alfa or beta when diluted in human plasma and heated at 37 °C (Fig. 1e).

### In vitro uptake study

Fabry patients-derived fibroblasts were incubated with different enzymes (10 μg/ml) overnight. Fibroblasts incubated with agalsidase alfa or beta had markedly increased intracellular α-gal A activities, and this uptake was nearly completely inhibited by M6P and was partially inhibited by mannan (Fig. 1f). Fibroblasts incubated with moss-aGal or high-mann moss-aGal had a significantly lower increment of α-gal A activities (Fig. 1f). This was consistent with little or no expression of MR in these cells (Fig. 1h,i). Despite the low uptake, lysosomal accumulation of Gb_3_ in Fabry patients’ fibroblasts was significantly decreased after treatment with moss-aGal or high-mann moss-aGal for 4 days (Fig. 1g), indicating that moss α-gal A enzymes are able to degrade the accumulated substrates in the lysosomes.

Endothelial cells are a major disease-relevant cell type in Fabry disease. We tested enzymatic uptake in Fabry patient-derived microvascular endothelial cells (IMFE1). IMFE1 cells originated from skin microvessels and were “immortalized” by ectopic expression of telomerase reverse transcriptase. These cells stably express many key markers and retain functional characteristics of microvascular endothelial cells (Shen et al 2007), and thus are a good in vitro model of human endothelium. IMFE1 cells were MR-positive (Fig. 1h,i). It is known that human dermal microvascular endothelia are MR-positive in vivo. However, MR expression in primary cultured microvascular endothelial cells decreased with subculture and was absent at passage 5 (Groger et al 2000), suggesting MR expression is prone to cellular senescence. The preserved expression of MR in IMFE1 cells and the low endogenous α-gal A activity make this cell line a unique tool for uptake studies. Expression of M6PR in IMFE1 was described previously (Marchesan et al 2012). After overnight incubation, moss α-gal A enzymes were efficiently taken up by IMFE1 cells (Fig. 1j). This uptake was predominantly blocked (~60-80 %) by mannan, suggesting it is mainly MR-mediated. Uptake of agalsidase alfa or beta by IMFE1 was mostly inhibited by M6P (~75-82 %).

In vitro uptake typically reaches a plateau phase after overnight incubation. To compare uptake rates in a dynamic phase, IMFE1 cells were incubated with the enzymes for a shorter time. Uptake of high-mann moss-aGal and agalsidase alfa was approximately linear for up to 3 h, with significantly higher uptake rate of high-mann moss-aGal than agalsidase alfa (Fig. 1k). Uptake of moss-aGal was remarkably higher than high-mann moss-aGal and agalsidase alfa after 1 h of incubation, and reached a plateau in 1–3 h (Fig. 1k). Immunoblot confirmed these results at the protein level (Fig. 1l).

Binding of different enzymes to IMFE1 cells was assessed. No α-gal A activity above background level was detected in cells incubated with high-mann moss-aGal or agalsidase alfa. Moss-aGal had significantly higher cellular binding than those enzymes, and this binding was blocked by mannan but not by M6P (Fig. 1m).

These results showed that in an assay system using cultured endothelial cells, which is likely more relevant to in vivo ERT than cultured fibroblasts, binding and uptake of moss α-gal A enzymes are more efficient than for agalsidase alfa, and this binding/uptake occurs through the MR. Since binding/uptake of moss-aGal was more efficient than high-mann moss-aGal, we selected the former for subsequent animal studies.

### Plasma pharmacokinetics

After infusion, moss-aGal was more rapidly cleared from circulation than agalsidase alfa when analyzed by enzyme activities (Supplementary Fig. 2a). To verify that the shorter plasma half-life of moss-aGal is due to more robust uptake by tissues rather than faster enzyme inactivation, enzymes in mouse plasma were also analyzed by immunoblot. α-Gal A protein levels in moss-aGal-infused mice at 5 and 10 min after infusion were significantly lower than in agalsidase alfa-injected mice, and there was a strong correlation between protein levels and enzyme activities in plasma (Supplementary Fig. 2b-d). Together with in vitro uptake study, these data suggested that administered moss-aGal is more efficiently taken up by vascular endothelial cells and other cell types compared to agalsidase alfa.

### Tissue and cellular distribution

**Fig. 2:**
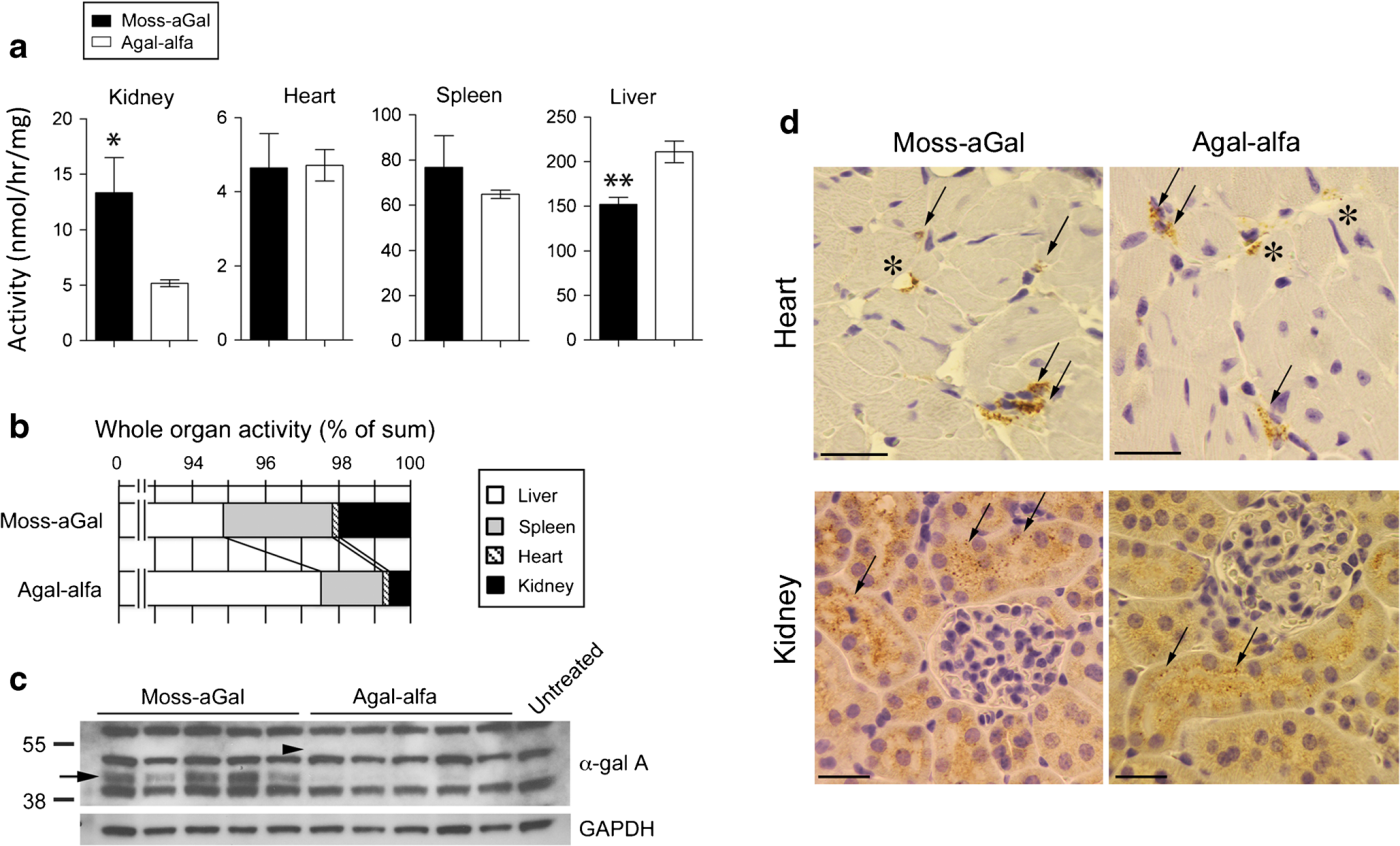
Tissue and cellular distribution of infused enzymes **a**-**c** Enzyme preparations were injected into Fabry mice, and α-gal A activities in the kidney, heart, spleen, and liver were measured 2 h post-injection. **a** Specific activities in organs. Data are presented as mean ± SEM (*n* = 5). *P < 0.05, **P < 0.01. **b** Activities in whole organs were calculated and data are presented as % of total activity recovered from four organs. **c** α-gal A protein in kidney homogenates detected by western blot. *Arrow*, specific α-gal A band in moss-aGal-injected mice. No detectable specific band was seen in agalsidase alfa-injected mice. *Arrowhead*, approximate position where agalsidase alfa band may migrate (based on findings shown in Fig. 1b). **d** Cellular distribution of infused enzymes in the heart and kidney was determined by immunohistochemistry (*n* = 2). Heart: asterisks indicate the blood vessels with immunostaining positive cells (most likely endothelial cells), and arrows indicate positive perivascular cells (presumably macrophages). Kidney: arrows indicate immunostaining positive tubular epithelial cells. Scale bar: 25 μm. Original magnification: 400×. Agal-alfa: agalsidase alfa

Biodistribution of moss-aGal and agalsidase alfa at 2 h post-injection was compared (Fig. 2a). Kidneys from moss-aGal-injected mice had significantly higher enzyme activities than those of agalsidase alfa-injected mice. The levels of moss-aGal and agalsidase alfa in the heart and spleen were comparable. The level of moss-aGal in the liver was significantly lower than that of agalsidase alfa. Activities per whole organs were calculated and ratios between different organs were compared (Fig. 2b). Among total recovered activities, 94.9 % of moss-aGal and 97.5 % of agalsidase alfa were delivered to the livers (*P* < 0.05). Kidneys of moss-aGal-injected mice had 1.96 % of total activity, which is significantly higher (*P* < 0.05) than that in agalsidase alfa-injected mice (0.58 %). Immunoblot confirmed the higher uptake of moss-aGal in the kidney compared to agalsidase alfa (Fig. 2c).

Cellular distribution of moss-aGal and agalsidase alfa was assessed by immunohistochemistry (Fig. 2d). Specific signals displayed granular cytoplasmic pattern, presumably reflecting lysosomal localization of the enzyme. Cellular localization of these two enzymes in the heart and kidney was essentially identical. In hearts, both enzymes were detected in capillaries and perivascular cells. In kidneys, specific staining was seen in cortical tubular epithelial cells for either enzyme. These results are consistent with distribution of agalsidase alfa described previously (Murray et al 2007).

### Tissue kinetics

Tissue kinetics of moss-aGal and agalsidase alfa were investigated (Supplementary Fig. 3). At 2 and 24 h post-injection, kidneys from moss-aGal-injected mice had significantly higher enzyme activities compared to agalsidase alfa-injected mice. However, activities were similar at 48 and 96 h. In the heart, there was no significant difference between the two forms of enzymes at 2 and 24 h; however, activities of moss-aGal were lower than agalsidase alfa at 48 and 96 h. In comparison to agalsidase alfa-injected mice, moss-aGal-injected mice had similar levels of activities in the spleen, and significantly lower activities in the liver at all time points analyzed. The half-lives of moss-aGal and agalsidase alfa in the kidney and heart ranged from 2 to 3 days. Moss-aGal had a ~25 % shorter half-life in both organs. The half-life of moss-aGal in the liver was significantly shorter compared to agalsidase alfa (24 vs. 57 h). The half-lives of both enzyme forms in the spleen were similar (~30 h). The relatively shorter half-life of moss-aGal in some organs is probably related to the lower carbohydrate content that may lead to increased susceptibility of the enzyme to proteolytic degradation in the lysosomes.

### Tissue Gb_3_ clearance

**Fig. 3:**
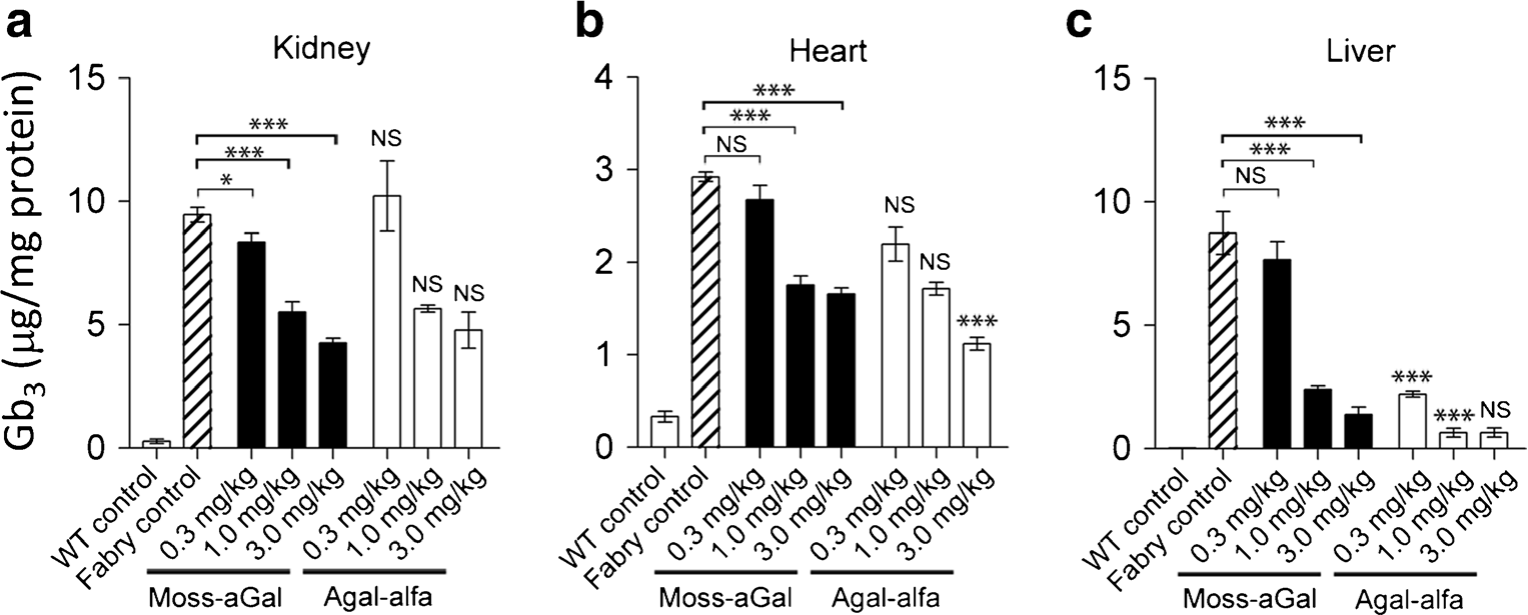
Efficacy of moss-aGal in clearing accumulated Gb_3_ in tissues. Gb_3_ contents in kidney (**a**), heart (**b**), and liver (**c**) were analyzed 7 days after a single infusion of either moss-aGal or agalsidase alfa at various doses. Data are presented as mean ± SEM (*n* = 4-5). *P < 0.05, ***P < 0.001. Statistical significance shown on top of each agalsidase alfa-injected group indicates difference between agalsidase alfa and the same dose of moss-aGal. Agal-alfa: agalsidase alfa

Efficacies of moss-aGal and agalsidase alfa in degrading accumulated Gb_3_ were compared at 7 days after a single intravenous injection of either enzyme in 6-month-old Fabry mice. Three different doses (0.3, 1, and 3 mg/kg) were tested. Both forms of enzymes reduced Gb_3_ in kidney, heart, and liver in a dose-dependent manner (Fig. 3a-c). Moss-aGal and agalsidase alfa had comparable efficacy in clearing Gb_3_ in the kidney and heart (Fig. 3a,b). In clearing liver Gb_3_, agalsidase alfa was more effective than moss-aGal at doses of 0.3 and 1 mg/kg (Fig. 3c). At a higher dose (3 mg/kg), these two enzymes led to similar liver Gb_3_ levels.

## Discussion

The present study provides new information on the relevance of the MR-dependent endocytic pathway in infusing therapeutic enzymes to treat systemic LSDs. Glycan structures and the results of in vitro studies indicated that uptake of moss-aGal is mediated by MR. The comparison of pharmacodynamic profiles of moss-aGal and agalsidase alfa in Fabry mice suggested that mannose-terminated enzymes can be as effective as M6P-harboring enzymes in the treatment of Fabry disease, and that M6P residues may not always be a prerequisite for ERT as generally believed. These findings may have important implications in developing new lysosomal enzymes in plant- and other non-mammalian cell-based (e.g., insect cell and yeast) expression systems, in which phosphorylation of mannosyl residues is either lacking (in case of plant and insect cells) or inadequate (in case of yeast). In yeast, mannose phosphorylation occurs, however, the phosphorylated sugars are capped by terminal mannose residues (Ballou 1990) that block the binding of M6PR to its ligands. Efforts have been applied to add M6P tag to plant-made enzymes (He et al 2012), or increase M6P content or expose the covered M6P in yeast-made enzymes (Chiba et al 2002; Tsukimura et al 2012). Although the role of MR in the therapeutic outcome of ERT can be α-gal A-specific, our study proposed a possibility that mannose-terminated enzymes produced from these non-mammalian cell systems, without M6P manipulations, may be sufficiently effective for some LSDs in which non-macrophage cells are affected.

However, our results also suggest that MR-dependent enzyme uptake can be largely influenced by mannose chain structures. In spite of increased terminal mannose residues, binding/uptake of high-mann moss-aGal to endothelial cells was significantly less efficient than moss-aGal. Similar findings have been noted by a previous study (Van Patten et al 2007), in which the MR binding of glucocerebrosidase with Man_9_GlcNAc_2_ was lower than that of Man_3_GlcNAc_2_. Therefore, it is likely that the affinity of core-type Man_3_GlcNAc_2_ for the MR is higher than that of high-mannose type N-glycans on lysosomal enzymes.

Fabry disease is one of the LSDs for which ERT has been most extensively investigated. Enzymes produced from mammalian cells, yeast, insect cells, and plants have been tested in in vitro and/or in vivo (Chen et al 2000a, b; Ioannou et al 2001; Chiba et al 2002; Tsukimura et al 2012; Kizhner et al 2015). The test of α-gal A produced in Chinese hamster ovary cells provided a prototype for the assessment of recombinant α-gal A in Fabry mice (Ioannou et al 2001). Recently, α-gal A produced from tobacco cells (PRX-102) was reported (Kizhner et al 2015). Like moss-aGal, PRX-102 is non-phosphorylated. However, this protein is chemically modified, resulting in a cross-linked dimer of PEGylated subunits. These modifications are associated with significant changes in protein characteristics, including different enzyme kinetics and dramatically prolonged circulation half-life (~10 h) compared with agalsidase alfa or beta. The uptake mechanism of PRX-102 remains to be elucidated. However, remarkably slow plasma clearance suggests that the uptake is not via M6PR- or MR-mediated endocytosis.

The present study demonstrated the therapeutic potential of moss-aGal as a new form of enzyme drug to treat Fabry disease. Moss-aGal protein is identical to its human counterpart with respect to amino acid sequence and dimeric structure. Moss-aGal has unique N-glycans that are predominantly core-type and are highly homogeneous. High degree of tri-mannosyl N-glycans was obtained on moss-aGal without using C-terminal vacuolar targeting signal. The latter is an often-used strategy in plant expression systems to achieve exposed mannosyl residues by sorting recombinant proteins to vacuolar compartments instead of Golgi complex. Downsides of this strategy are that the additional amino acids will remain in the final protein product and purification of the protein requires extraction from a whole cell lysate.

Due to depletion of plant-specific glycosyltransferases in the host strain, moss-aGal does not have α-(1,3)-fucose and β-(1,2)-xylose residues, hence avoiding potential immunological reaction against these plant-specific sugar chains. In general, development of antibodies to the infused agalsidase alfa or beta is common in enzyme therapy for Fabry patients, especially in males, which may affect the efficacy and safety of the treatment (Deegan 2012). Sugar chains exert important roles in modulating the antigenicity of therapeutic proteins (Costa et al 2014), thus the distinct glycosylation profile of moss-aGal from that of agalsidase alfa or beta will likely lead to a different immunological response in the human body. This should be investigated in future studies.

The uptake of moss-aGal by IMFE1 cells was more efficient than that of agalsidase alfa. Given that endothelial cells may play roles in the pathophysiology of vasculopathy and other manifestations in Fabry disease, the better delivery to vascular endothelial cells could be advantageous. Uptake of agalsidase alfa and beta by endothelial cells involved both MR and M6PR, with the latter as preferential pathway. The less efficient endothelial uptake of agalsidase alfa compared to moss-aGal is probably due to lower abundance of M6PR in these cells relative to MR. The low expression of plasma membrane M6PR in human endothelial cells demonstrated by Marchesan (Marchesan et al 2012) supports this possibility. However, discrepancies exist between the same study by Marchesan et al and ours with respect to uptake mechanism of agalsidase alfa/beta in endothelial cells. Their study found no M6PR-mediated binding/uptake and little lysosomal delivery of α-gal A in human endothelial cells. By contrast, data from our present and previous studies suggested that M6PR pathway is the dominant contributor for uptake of agalsidase alfa/beta in IMFE1 cells, and that the enzyme is delivered into lysosomes, as evidenced by the robust clearance of lysosomal Gb_3_ in enzyme-treated cells (Shen et al 2007). These discrepancies may be due to methodological differences. For example, Marchesan and coworkers used fluorescence detection of labeled α-gal A for uptake and lysosomal delivery of the enzyme, while we used enzyme assay and Gb_3_ immunocytochemistry.

Nevertheless, it should be noted that vascular endothelium is readily accessed by intravenously administered α-gal A (Eng et al 2001a, b; Schiffmann et al 2001). Cardiomyocytes, podocytes, and peripheral neurons represent the major hard-to-reach cell types in current ERT for Fabry disease. It remains unclear whether moss-aGal offers improved delivery to these cell types compared with existing enzymes, because we could not detect any infused enzyme in cardiomyocytes and podocytes by immunohistochemistry, possibly due to lower sensitivity of the method.

Relative to agalsidase alfa, targeting of moss-aGal to the kidney was significantly enhanced and delivery to the liver was significantly reduced. The mechanism for this differential tissue distribution is unclear. One possibility is that the lower uptake of moss-aGal by the liver might contribute to relatively increased uptake by the kidney. However, this cannot explain the absence of relative increased uptake of moss-aGal by the heart and spleen compared to agalsidase alfa. Thus, it is likely that there is a more specific mechanism that targets moss-aGal preferentially to the kidney. Because the infused enzyme was only detected in cortical tubules by immunostaining, a large part of α-gal A activity in the kidney must have been from the tubules, suggesting that the higher renal distribution of moss-aGal may be due to the higher uptake of tubular cells. However, renal tubules were not reported to express MR (Linehan et al 1999). A potential interpretation is that tubular cells express MR, but at a relatively low level that could not be detected, or that they express other receptor(s) that mediate endocytosis of mannose-terminated glycoproteins. The presence of such unidentified receptor(s) that have MR-like binding activity has been reported in murine spleens and lymph nodes (Linehan et al 1999). Another possibility is endocytosis through other receptors that recognize non-carbohydrate ligands. It is known that megalin and/or sortilin work as α-gal A receptors in tubular cells, podocytes and glomerular endothelial cells (Christensen et al 2007; Prabakaran et al 2011, 2012). Further studies are needed to test whether moss-aGal has higher affinity for these receptors compared to mammalian cell-produced enzymes, and thus leads to better renal distribution.

Our study also provided new insight into endocytic pathways for the phosphorylated form of α-gal A. As mentioned, both M6PR and MR mediate delivery of agalsidase alfa in vitro, thus it is difficult to determine which receptor pathway is more responsible for the biodistribution of this enzyme in target organs. Despite markedly different sugar chains, cellular localization of agalsidase alfa and moss-aGal in the heart and kidney was surprisingly similar. Storage clearance efficacy in these organs was similar as well. In other words, compared to a completely non-phosphorylated enzyme, M6P residues in agalsidase alfa did not lead to a wider distribution and more complete Gb_3_ clearance as one might expect. These findings suggested that the MR pathway might play a more important role than M6PR in targeting agalsidase alfa to the heart and kidney. These findings are different from those of previous studies that suggested the importance of M6P in delivery of β-glucuronidase and acid α-glucosidase (Sands et al 2001; Zhu et al 2009). The discrepancy between α-gal A and these enzymes suggests that the impact of M6P in ERT can be enzyme-specific.

## Electronic supplementary material

ESM 1 (DOCX 1261 kb)
