# Supplementary material for: Mannose receptor-mediated delivery of moss-made α-galactosidase A efficiently corrects enzyme deficiency in Fabry mice
Source: J Inherit Metab Dis. 2015 Aug 27;39:293–303. doi: 10.1007/s10545-015-9886-9 (PMC4754329; doi:10.1007/s10545-015-9886-9)
Supplement: Supplementary file 1 — (DOCX 1261 kb) [file 10545_2015_9886_MOESM1_ESM.docx]

**Supplementary Materials**

**Materials and Methods:**

**Enzyme production and purification:**

The moss-aGal production strain was cultivated for 4 weeks in a 20 L disposable bag (Cellbag 20, GE Healthcare, Germany) placed in a Wave™ Reactor Rocker (BioWave 20 SPS, Wave Biotech AG, Switzerland). The cultivation parameters were: 25 to 30 rpm rocking rate, 8° angle; SM07 medium (100 mM NaCl, 6.6 mM KCL, 2.0 mM MgSO_4_ x 7H_2_O, 1.8 mM KH_2_PO_4_, 20.4 mM Ca(NO_3_)_2_ x 4H_2_O, 0.05 mM Fe Na-EDTA, 4.9mM MES, 0.1 % (w/v) PEG4000, 100.26 µM H_3_BO_3_, 0.11 µM CoCl_2_ x 6H_2_O, 0.1 µM CuSO_4_ x 5H_2_O, 5 µM KI, 85.39 µM MnCl_2_ x 4H_2_O, 1.03 µM Na_2_MoO_4_ x 2H_2_O, 0.11 mM NiCl_2_ x 6H_2_O, 0.04 Na_2_SeO_3_ x 5H_2_O, 0.039 Zn-acetate x 2H_2_O); 25°C temperature; gassing of 0.3 L x min^-1^ pressured air supplemented with 2% to 4% CO_2_ and illumination at 130 to 310 µE x m^-2^ x s^-1^, 24 h light per day, delivered from light panels equipped with Osram FQ 24W 840 HO, Lumilux Cool White. The medium was additionally supplemented with 1000x Nitsch vitamin mixture (Nitsch vitamin mixture, Duchefa, Netherlands) according to manufacturer’s instructions. The pH-value of the fermentation was controlled automatically at pH 5-6 through titration with 0.5 M H_2_SO_4_ and 0.5 M NaOH with help of WAVEPOD I (GE Healthcare) in combination with Pump20 (GE Healthcare).

At the end of cultivation, the culture broth was clarified through 3 steps filtration cascade: 1) removal of moss through cake filtration in customized PP filtration housing (Grosse, T., Niederkrüger, H. and Schaaf, A. 2014; EP 2687592 A1: Filtration of cell culture supernatants. http://www.google.com/patents/EP2687592A1?cl=en) equipped with Zetaplus (01SP B3002, 3M, Germany), 2) depth filtration through a double layer Scale-Up Capsule (E0340FSA60SP03A, 3M, Germany) and 3) a sterile filtration (Millipore Express^TM^ Plus, 0.22 µm, Millipore, Germany). The clarified filtrate was concentrated and rebuffered using tangential flow filtration (Pall Centramate 500S, 30kDa cutoff cellulose membrane). After a series of 3 chromatographic steps (Butyl-650M, DEAE, S), the purified moss-aGal and high-mann moss-aGal were concentrated to ~0.5 mg/ml and were stored at 4°C until use.

**Glycan analysis:**

Glycan analysis of moss-aGal and agalsidase alfa was done by Protagen Protein Services (Dortmund, Germany) using HILIC-UPLC-MS. In short, N-glycans were released from the protein enzymatically using PNGase F. After cleanup and desalting, isolated glycans were labeled using 2-aminiobenzamide (2-AB). Labeled glycans were separated on a ACQUITY UPLC BEH Glycan (2.1x100mm) column using a linear gradient of 78% to 55.9% B (buffer A: 100mM ammoniumformate pH4.5, buffer B: acetonitrile) in 38.5min at 60°C with a flow rate of 0.5ml/min. Signals of eluting glycans were recorded by a fluorescence detector (excitation at 330nm, emission at 420nm). The assignment of fluorescence peaks to the respective glycans was done using recorded m/z values (Xevo-QTOF MS, Waters) and MassLynx software (Version 4.1, Waters).

Glycan analysis of high-mann moss-aGal was performed as follows. About 25 µg of high-mann moss-aGal was reduced (15mM DTT), carbamidomethylated (55 mM iodoacetamide) and acetone precipitated (acetone:aqueous phase 4:1). The pellet was dissolved in 0.1M ammonium bicarbonate buffer and digested with either trypsin or chymotrypsin (both sequencing grade, Roche) for 12 hours at 37°C. About 3 µg of each digest was loaded on a BioBasic C18 column (BioBasic-18, 150 x 0.32 mm, 5 µm, Thermo Scientific) using 60 mM ammonium formate buffer as the aqueous solvent. A gradient from 3 to 75 % acetonitrile was developed over 25 min at a flow rate of 6 µL/min. Detection was performed with a Waters Q-TOF Ultima mass spectrometer equipped with the standard ESI source in the positive ion mode. Data analysis was performed manually with MassLynx4.0.

**Michaelis–Menten kinetics:**

Moss-aGal or agalsidase alfa were incubated with 8 different concentrations of artificial substrate 4-nitrophenyl α-D-galactopyranoside at 37°C in 100 mM Na-phosphate, 0.4% bovine serum albumin at pH 4.8. After 10 min the reaction was stopped with 200 mM borate (pH 9.8) and product formation was measured at 410 nm. All measurements were done in triplicates. Product concentration was calculated using ε=18.5 ml*mmol^-1^*cm^-1^. Km and Vmax were calculated using Graph Pad Prism 6.07.

**In vitro thermostability:**

Enzymes were diluted in plasma obtained from a healthy individual and were heated at 37°C for indicated time lengths. To keep neutral pH, HEPES were added to the plasma at final concentration of 20mM. After heating, α-gal A activities were measured.

**SDS-PAGE and western blot:**

Samples were denatured in LDS sample buffer (Invitrogen, Carlsbad, CA) at 70°C for 10min in the presence of 2.5% 2-mercaptoethanol. NuPAGE Bis-Tris 4-12% or 10% gels (Invitrogen) were used for protein separation. Western blot was performed as described previously ([Shen et al 2008](#_ENREF_2)). Non-reducing conditions were used for western blot analysis of MR. Primary antibodies used were rabbit polyclonal antibody to human α-gal A (Shire Human Genetic Therapies, Cambridge, MA), mouse monoclonal antibody to mannose receptor (clone 15-2, Abcam, Cambridge, MA) and goat polyclonal antibody to GAPDH (Santa Cruz Biotechnology, Santa Cruz, CA). The α-gal A protein levels were quantified by densitometry using ImageJ software.

**Immunofluorescence of cultured cells:**

Fluorescence immunostaining was performed as described previously ([Shen et al 2008](#_ENREF_2)). The primary antibodies used were mouse monoclonal antibodies to Gb_3_ (Seikagaku, Tokyo, Japan) and mannose receptor (clone 15-2, Abcam). The cells were counterstained with DAPI.

**Immunohistochemistry:**

Moss-aGal or agalsidase alfa was injected via tail-vein at a dose of 1 mg/kg body weight (n=2 each). Heart and kidney were harvested 1 day after enzyme infusion. Untreated female Fabry mouse tissues were used as negative controls. Tissues were fixed in formalin, embedded in paraffin, and 5-micron sections were made. Immunohistochemistry was performed by Histopathology and Tissue Shared Resource in Georgetown University (Washington, D.C.). In brief, after heat-induced epitope retrieval in citrate buffer, sections were treated with 3% hydrogen peroxide and 10% normal goat serum, and were incubated with rabbit polyclonal antibody to human α-gal A (Shire). After incubation with HRP-labeled secondary antibody, signals were detected by DAB chromogen, and the sections were counterstained with hematoxylin. Signal specificity was verified with control staining, in which the primary antibody incubation was omitted. Compared to light and diffuse non-specific staining in untreated controls, specific signal displayed a granular cytoplasmic pattern, which is consistent with findings from a previous study ([Murray et al 2007](#_ENREF_1)).

**Reference:**

Murray GJ, Anver MR, Kennedy MA, Quirk JM, Schiffmann R (2007) Cellular and tissue distribution of intravenously administered agalsidase alfa. *Mol Genet Metab* 90: 307-312.

Shen JS, Meng XL, Moore DF, et al (2008) Globotriaosylceramide induces oxidative stress and up-regulates cell adhesion molecule expression in Fabry disease endothelial cells. *Mol Genet Metab* 95: 163-168.

**Supplemental Figure 1**

**
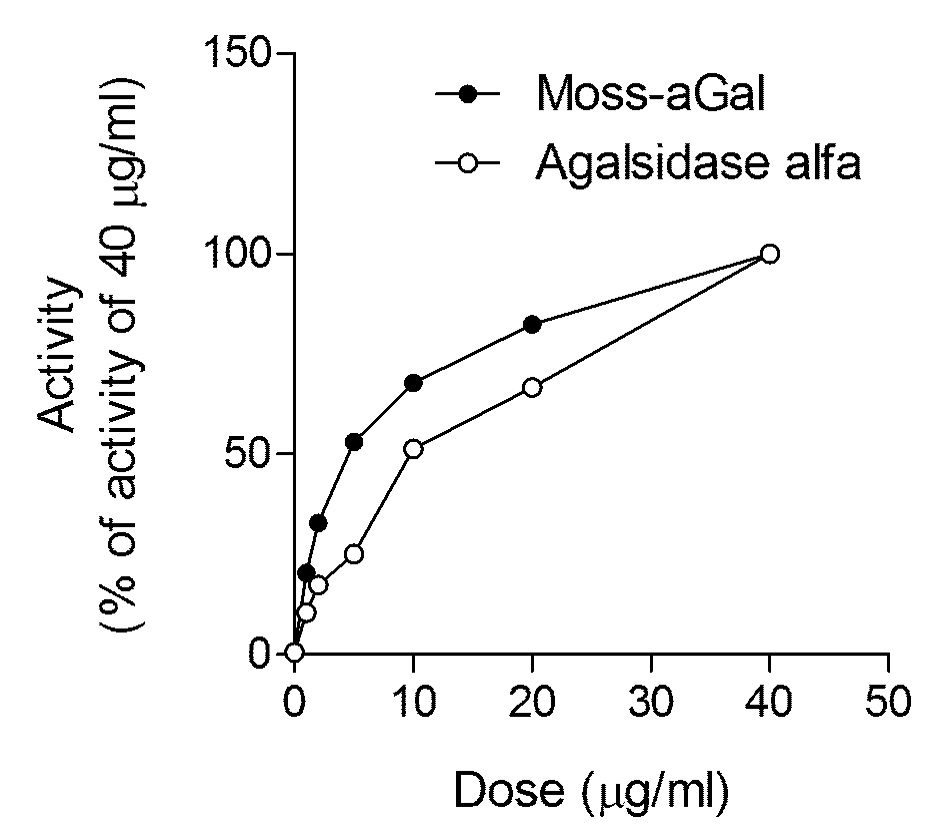
**

**Supplemental Figure 1. Uptake of α-gal A in IMFE1 cells at various concentrations**

IMFE1 cells were incubated with various concentrations of moss-aGal and agalsidase alfa (n=2). Intracellular α-gal A activities were measured 5 hours after incubation. Data are means of duplicates.

**Supplemental Figure 2**


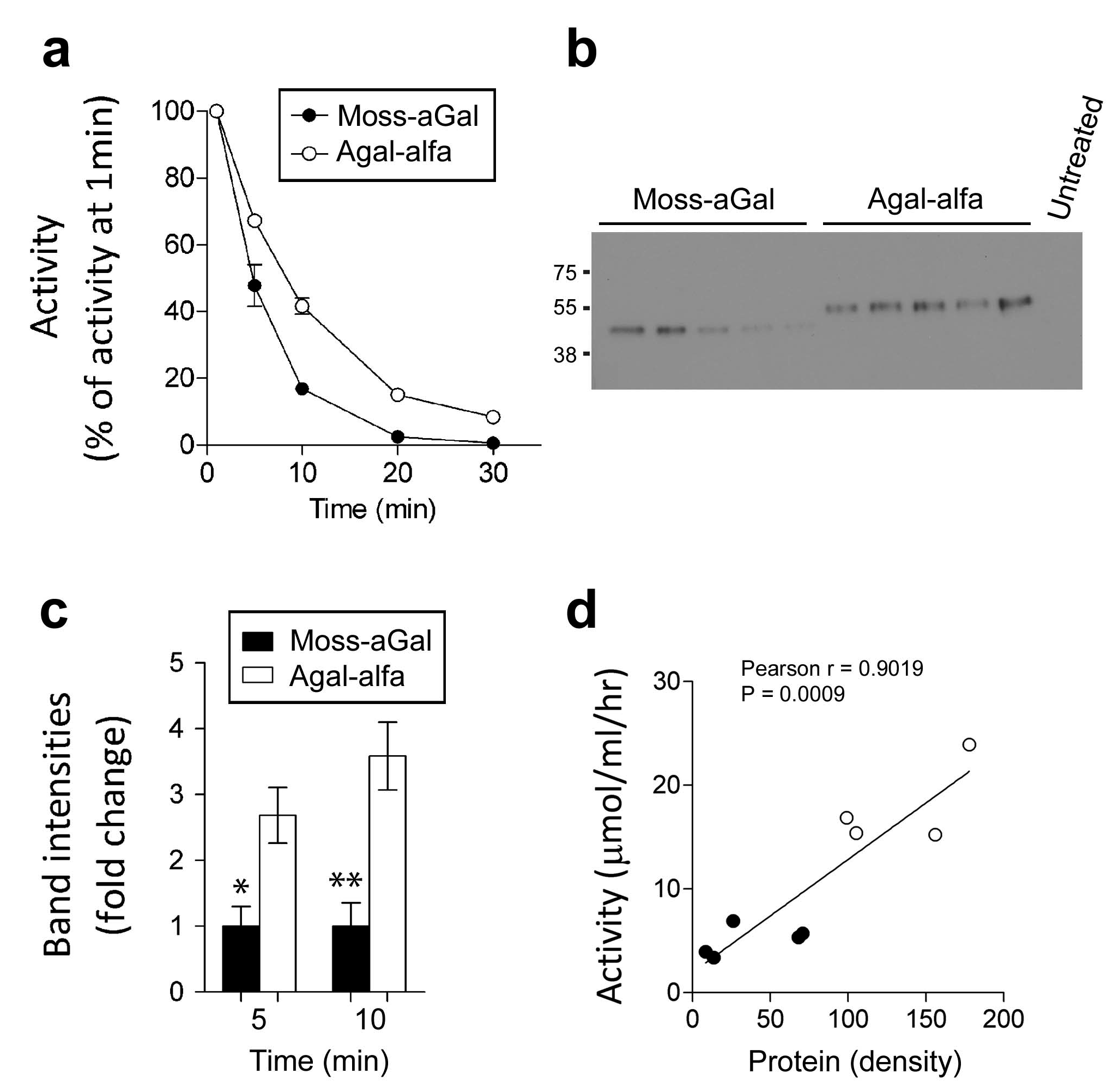


**Supplemental Figure 2. Plasma pharmacokinetics**

(**a**) Plasma clearance of infused moss-aGal and agalsidase alfa. Blood samples were collected at 1, 5, 10, 20 and 30 min after injection. α-Gal A activity in plasma was measured. Half-lives of moss-aGal and agalsidase alfa in circulation were 3.6 and 7.9 min respectively. (**b**) Western blot for α-gal A in plasma at 10 min after infusion. (**c**) α-Gal A protein amounts in plasma at 5 and 10 min after infusion. Western blot bands intensities were analyzed by densitometry. According to the higher reactivity of the antibody to moss-aGal (see main text), the intensities of moss-aGal bands were corrected by a factor of 2.14. (**d**) Correlation between α-gal A protein amounts and enzymatic activities in plasma at 10 min after injection. Data in (**a**) and (**c**) are presented as mean ± SEM (n=4-5). *P<0.05, **P<0.01. Agal-alfa: agalsidase alfa.

**Supplemental Figure 3**


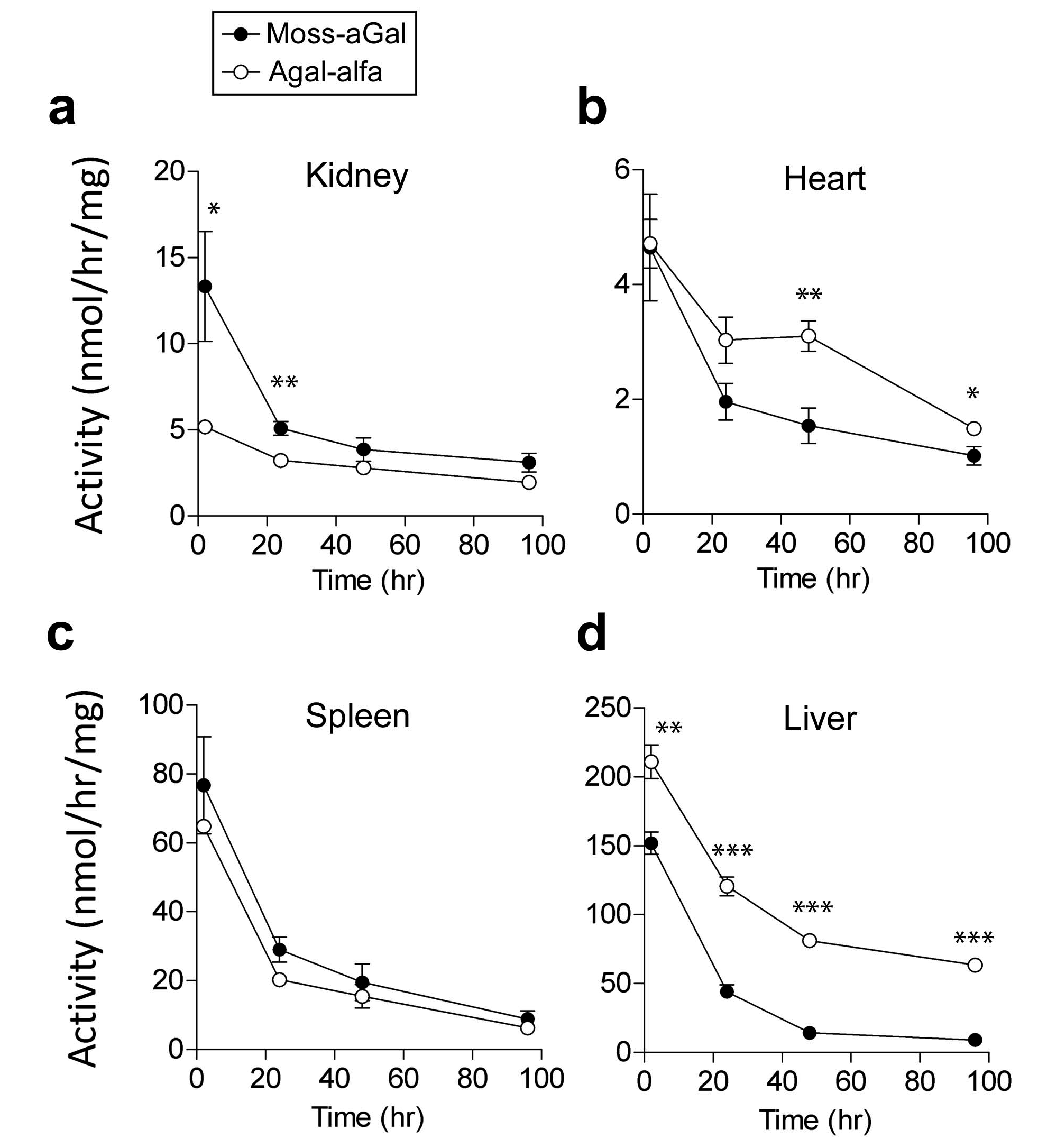


**Supplemental Figure 3. Tissue kinetics of infused enzymes**

Enzyme preparations were injected into Fabry mice, and α-gal A activities in kidney (**a**), heart (**b**), spleen (**c**) and liver (**d**) were measured at 2, 24, 48 and 96 hours post-injection. Data are presented as mean ± SEM (n=4-5). *P<0.05, **P<0.01, ***P<0.001. Agal-alfa: agalsidase alfa.
